# Establishing equivalent diabetes in male and female Nos3‐deficient mice results in a comparable onset of diabetic kidney injury

**DOI:** 10.14814/phy2.14197

**Published:** 2019-09-19

**Authors:** Lifang Tian, David J. Nikolic‐Paterson, Greg H. Tesch

**Affiliations:** ^1^ Department of Nephrology Monash Medical Centre Clayton Victoria Australia; ^2^ Department of Nephrology The Second Affiliated Hospital of Xi'an Jiaotong University Xi'an Shaanxi China; ^3^ Centre for Inflammatory Diseases Monash University Clayton Victoria Australia

**Keywords:** Diabetic kidney disease, diabetic nephropathy, endothelial nitric oxide synthase, glomerulosclerosis, nitric oxide synthase 3

## Abstract

Clinical studies indicate that sex differences exist in susceptibility for developing diabetic kidney disease (DKD), supporting the need to examine both sexes in animal studies of DKD. Streptozotocin (STZ) is commonly used in male mice to induce diabetes and DKD. However, females are not normally included because their sex hormones partially protect them from STZ‐induced islet injury and consequent diabetes. To address this issue, we identified a strategy to induce comparable diabetes in male and female mice using STZ and determined whether both sexes develop equivalent renal injury. Male and female mice lacking the gene for endothelial nitric oxide synthase (*Nos*3‐/‐) were made diabetic with five or six low‐dose STZ injections, respectively. Groups of male and female mice with equivalent hyperglycemia at week 3 after STZ were assessed for DKD at week 8. STZ‐treated male and female *Nos*3‐/‐ mice maintained comparable hyperglycemia between weeks 3 and 8 had an equivalent increase in HbA1c levels and comparable hypertension. Urine albumin/creatinine levels were elevated eightfold in mice of both sexes at week 8, accompanied by an equivalent loss of podocytes. In diabetic males and females, plasma cystatin C levels and glomerular collagen deposition were similarly increased. Kidney mRNA levels of proinflammatory and profibrotic markers and kidney injury molecule‐1 (KIM‐1) were equally elevated in males and females, indicating comparable kidney injury. This study shows that equivalent diabetes induces a comparable onset of DKD in male and female *Nos*3‐/‐ mice, demonstrating that it is possible to include males and females together in studies of DKD.

## Introduction

Diabetic kidney disease (DKD) is the most prevalent cause of end‐stage kidney disease (ESKD) affecting both men and women (Harjutsalo and Groop, 2014). Several studies have indicated that diabetic males have an increased risk of developing albuminuria and renal function impairment, although this does vary with age and is confounded by hypertension and obesity (Harjutsalo et al. 2011; Neugarten and Golestaneh, 2013; Clotet et al. 2016). Gender differences in DKD are thought to be related to levels of the sex hormones 17*β*‐estradiol and testosterone (Dixon and Maric, 2007; Xu et al. 2008; Catanuto et al. 2009), but are not clearly defined (Diamond‐Stanic et al. 2012). Some experimental studies suggest that these sex hormones can influence the progression of diabetic nephropathy by regulating TGF‐*β* signaling, macrophage infiltration, or components of the renin angiotensin system (Diamond‐Stanic et al. 2012; Clotet et al. 2016). These findings indicate that optimal clinical treatment of patients with DKD may require a better understanding of potential sex differences in disease progression associated with age and other comorbidities. Some insight into potential sex differences in the development of DKD has been seen in animal model studies comparing males and females with streptozotocin (STZ)‐induced type 1 diabetes; however, these comparisons are rare and have involved a short duration of type 1 diabetes (2 weeks) (Lovegrove et al. 2004; Wells et al. 2005) or have not monitored the progression of diabetes after hyperglycemia was established (Yamaleyeva et al. 2012; de Alencar Franco Costa et al. 2015).

Most animal studies of DKD are performed in males. This is predominantly because females have a lower susceptibility to developing diabetes due to 17*β*‐estradiol protecting pancreatic *β* cells from oxidative injury (May et al. 2006). Consequently, female rodents are generally more resistant to induction of pancreatic islet injury by STZ, which is routinely used to create models of type 1 diabetes. Because the same dosage of STZ induces different levels of hyperglycemia in male and female rodents (May et al. 2006), it has been difficult to directly compare type 1 DKD in experimental animals. Furthermore, female rodents also appear more resistant to type 2 diabetes, because food intake is usually higher in males which is associated with earlier increases in blood glucose and earlier declines in insulin (Chow et al. 2004; Ohta et al. 2014). These sex differences have resulted in a failure to adequately compare the development of DKD in males and females in experimental animal models and to compare the outcomes of therapeutic interventions in both sexes.

Mice deficient in endothelial nitric oxide synthase (*eNOS/Nos3)* are widely used as a model to examine the development of DKD and the impact of novel intervention therapies (Takahashi and Harris, 2014; Tesch et al. 2015). *Nos3‐/‐* mice are moderately hypertensive and develop a slowly progressive CKD which is markedly exacerbated by the presence of type 1 or type 2 diabetes (Zhao et al. 2006; Nakagawa et al. 2007). Diabetic *Nos3‐/‐* mice develop substantial albuminuria, kidney inflammation, nodular glomerulosclerosis, and renal function impairment which resembles progressive DKD in patients (Zhao et al. 2006; Nakagawa et al. 2007; Takahashi and Harris, 2014). This model meets the pathological feature requirements put forward by NIDDK sponsored Diabetes Complications Consortium (www.diacomp.org) which identifies a models' suitability for experimental studies of DKD. However, one limitation of using *Nos3‐/‐* mice is that they have reduced breeding efficiency compared to wild‐type strains. Their smaller litter sizes (typically 4–6) are due to *Nos3* deficiency causing alterations in the estrous cycle, ovulation, and prenatal development (Drazen et al. 1999; Kulandavelu et al. 2013).

There is growing acceptance that it is important to perform animal model studies of DKD in both sexes to determine whether disease pathways can differ and if therapeutic targeting strategies are equally effective in both sexes. In support of this, the US National Institute of Health now expects that investigators include potential sex differences as a biological variable which will be incorporated into experimental design, analyses, and reporting (http://orwh.od.nih.gov/research/strategicplan/). Therefore, to help address this sex issue, and to facilitate the experimental use of both sexes of *Nos3‐/‐* mice, we sought to establish whether inducing equivalent diabetes results in a comparable onset of DKD in male and female *Nos3‐/‐* mice.

## Materials and Methods

### Animal models

*Nos3‐/‐* mice on the C57BL/6 background were obtained from Jackson Laboratories (Bar Harbor, ME) and bred under pathogen‐free conditions at the Monash Medical Centre Animal Facility (Clayton, Australia). At 8 weeks of age, *Nos3‐/‐* mice were given intraperitoneal injections of STZ (5 × 55 mg/kg/day; Sigma, St Louis, MO). Females were given one additional injection of STZ 1 week later. Groups of male and female mice (*n* = 8) with equivalent diabetes (fasting blood glucose > 16 mmol/L at 3 weeks after the fifth STZ injection) were followed for 8 weeks. Age‐matched nondiabetic male and female *Nos3‐/‐* mice (*n* = 8) were used as controls. Blood glucose (measured by a tail vein sample) and body weight were measured weekly after a 3 h fast (0800–1100) and mice with fasting glucose levels >30 mmol/L were given 0.5 units of protaphane insulin (Novo Nordisk, Sydney, Australia) subcutaneously three times a week to maintain body weight. Urine was collected preexperiment and at week 8 of the experiment to assess urine albumin excretion. Glycated hemoglobin (HbA_1_c) was measured from blood samples taken at week 8. Kidneys collected at week 8 were weighed and fixed in 4% (vol/vol) formaldehyde, 2% (wt/vol) paraformaldehyde‐lysine‐periodate (PLP) or methylcarn solution, or snap‐frozen, and stored at −80°C.

### Biochemistry

Fasting blood glucose was measured from tail blood by glucometer (Medisense, Abbott Laboratories, Bedford, MA). Urine was collected from mice housed in metabolic cages for 6 h. Heparinized whole blood was collected by cardiac puncture in anesthetized animals for analysis of HbA_1_c and isolation of heparinized plasma. Urine creatinine levels were determined by the Jaffe rate reaction method. ELISA kits were used to assess levels of urine albumin (Bethyl Laboratories, Montgomery, TX) and plasma cystatin C (R & D Systems, Minneapolis, MN). Albuminuria was measured by calculating the urine albumin/creatinine ratio (ACR). HbA1c was measured by DCA Vantage Analyzer (Siemens, Camberley, UK).

### Blood pressure analysis

Systolic blood pressure (SBP) was measured in conscious mice by tail‐cuff plethysmography (IITC Life Science, Woodland Hills, CA) (Tesch et al. 2015) during the final week of experimentation. Mice were trained twice weekly for 3 weeks prior to experimental readings. At each recording, mice were acclimatized to a preheated chamber (30°C) for 15 min and the pressure readings were recorded over three consecutive manual inflation‐deflation cycles to obtain an average.

### Antibodies

The primary antibodies used in this study were: goat anti‐collagen IV (1:200 dilution, Southern Biotechnology, Birmingham, AL), and rabbit monoclonal anti‐Wilm's Tumor 1 (WT‐1) antigen (Ab89901, 1:400 dilution, Abcam, Cambridge, United Kingdom).

### Immunohistochemistry

Formalin‐fixed sections (2 *μ*m) were stained with Periodic acid–Schiff (PAS) to assess structure and counterstained with hematoxylin to identify nuclei. Immunostaining for WT‐1 or collagen IV was performed on 4 *μ*m‐paraffin embedded sections which were fixed in methylcarn solution. For immunostaining, sections were treated with 20% rabbit serum or 20% goat serum for 30 min and then incubated with primary antibody in 3% BSA overnight at 4°C. Sections were then placed in 0.6% hydrogen peroxide in methanol for 20 min to inactivate endogenous peroxidase. Bound primary antibodies were detected using a standard ABC‐peroxidase system: avidin‐biotin block, biotinylated antibodies (rabbit anti‐goat IgG, rabbit anti‐rat IgG, or goat anti‐mouse IgG), and ABC‐peroxidase (Vector Laboratories, Burlingame, CA). Sections were developed with 3,3‐diaminobenzidine (Sigma) to produce a brown color. Normal goat serum or isotype‐matched irrelevant IgG was used as negative controls.

### Quantitation of immunohistochemistry

The number of WT‐1+ podocytes was counted in 30 hilar glomerular cross‐sections (gcs) per animal (×400). Glomerular collagen IV staining was quantitated by computer image analysis (Image‐Pro Plus, Media Cybernetics, Silver Spring, MD) in 30 hilar gcs (×400), and expressed as the percentage of glomerular area stained. All scoring was performed on blinded slides.

### Real‐time PCR

A section of kidney cortex was snap‐frozen and stored at −80°C until use. RNA was extracted from frozen tissues using the RiboPure RNA isolation kit (Ambion, Austin, TX). RNA was reverse transcribed with random hexamer primers and the Superscript III kit (Invitrogen, Carlsbad, CA). Real‐time RT‐PCR was performed on a StepOne machine (Applied Biosystems, Mulgrave, VIC, Australia) with thermal cycling conditions of 50°C for 2 min, 95°C for 10 min, followed by 40 cycles of 95°C for 15 sec and 60°C for 1 min. Primers and FAM‐labeled MGB probes for detection of CD68, TNF‐*α*, CCL2, KIM‐1, TGF‐*β*1, fibronectin, and collagen I and IV have been described previously (Tesch et al. 2015). Primers and VIC‐labeled probes for 18S ribosomal RNA were purchased from Applied Biosystems. All amplicons were normalized against the 18S RNA internal control (Applied Biosystems). The relative amount of individual mRNA species was calculated using the comparative Ct method. Real‐time PCR data were recorded as fold differences relative to nondiabetic females which was set to a value of 1.0.

### Statistical analysis

Because we established equivalent diabetes in male and female mice, we assessed statistical differences in renal outcomes between sexes by one‐way ANOVA with Tukey's multiple comparison posttest. Graphed data were recorded as mean ± SEM with *P* < 0.05 considered significant. All analyses were performed using GraphPad Prism 7.0 (GraphPad software, San Diego, CA).

## Results

### Effect of sex on development of diabetes and hypertension

**Figure 1:**
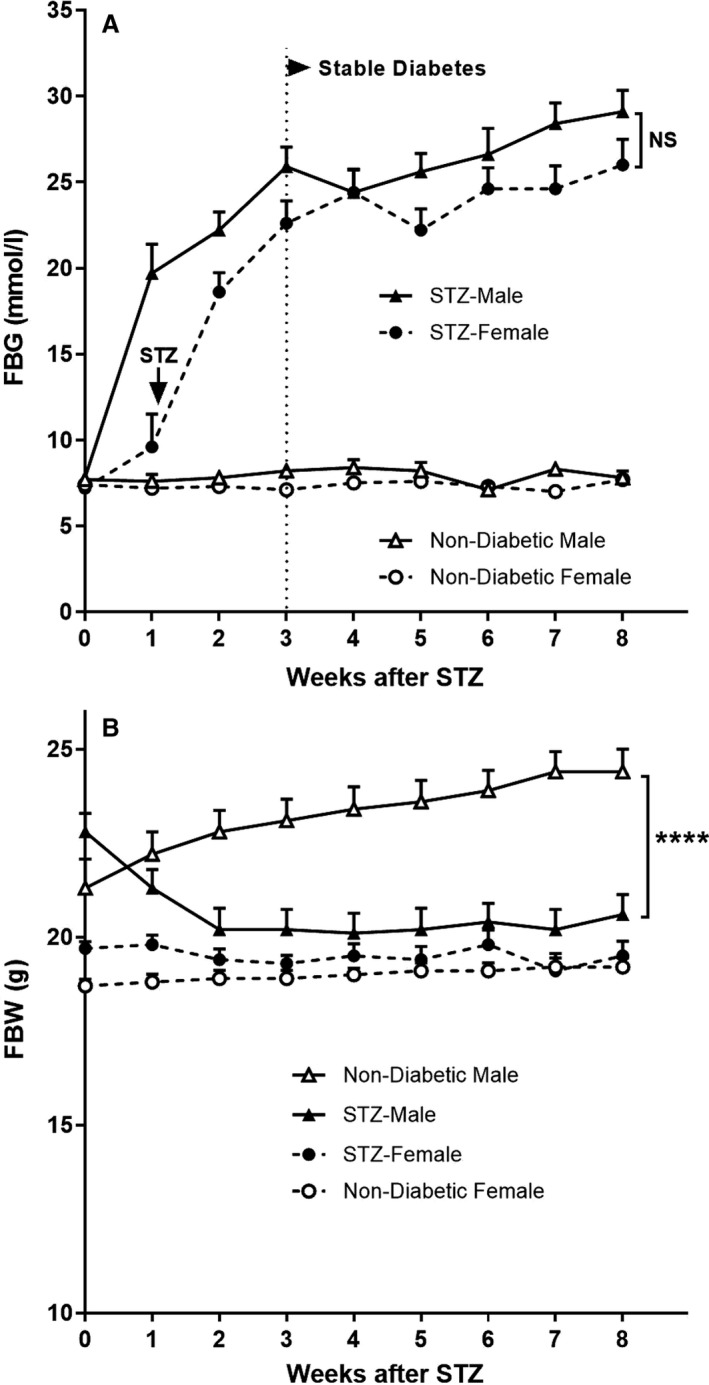
Effect of sex on the development of diabetes in *Nos3‐/‐* mice. Age‐matched groups of female and male *Nos3‐/‐* mice were given STZ (diabetic mice) or no STZ (nondiabetic controls) and monitored weekly for (A) fasting blood glucose (FBG) and (B) fasting body weight (FBW). Data = mean ± SEM, *n* = 8. ****P < 0.0001. NS = not significant.

**Figure 2:**
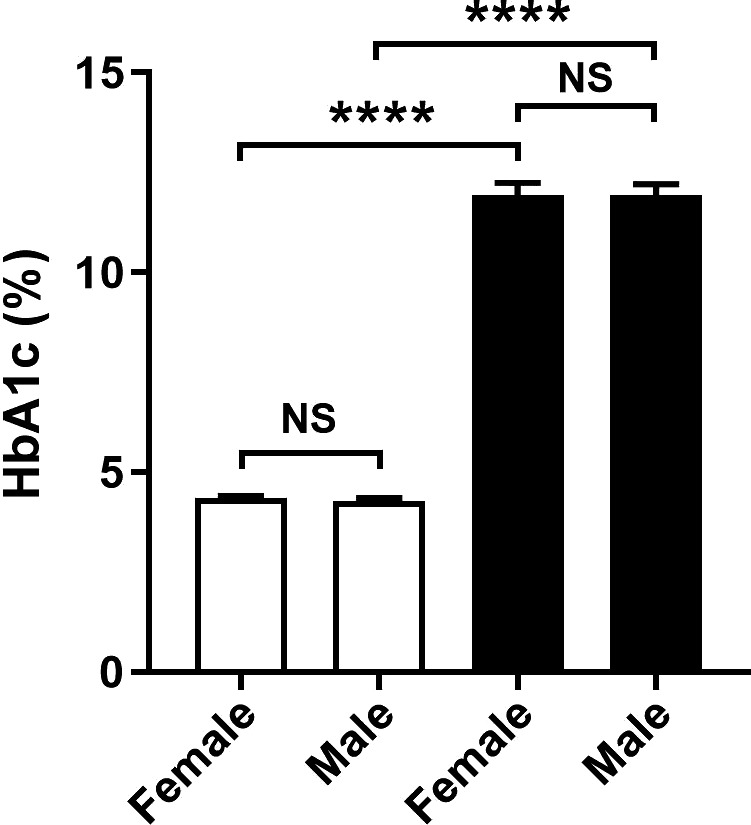
Effect of diabetes on glycated hemoglobin levels in *Nos3‐/‐* mice. At week 8 of the experiment, diabetic *Nos3‐/‐* mice (filled bars) and nondiabetic *Nos3‐/‐* mice (unfilled bars) of both sexes were assessed for levels of glycated hemoglobin (% HbA1c) as a measure of blood glucose control. Data = mean ± SEM, *n* = 8. ****P < 0.0001. NS = not significant.

Fasting blood glucose levels were significantly elevated in male mice at 1 week after the fifth STZ injection and continued to rise steadily before reaching a plateau at week 3 (Fig. 1A). In comparison, fasting blood glucose levels in female mice were not significantly raised at week 1 after the fifth STZ injection (Fig. 1A), and so were given an additional sixth injection of STZ. A week after the sixth STZ injection (week 2 of the experiment), fasting blood glucose levels in female mice were significantly elevated and continued to rise before reaching a plateau at week 3. Between weeks 3 and 8 of the experiment, the fasting blood glucose levels remained similar in male and female mice. Analysis of blood at week 8 found that levels of HbA1c were the same in male and female diabetic mice (Fig. 2), demonstrating that equivalent diabetes had been achieved in both sexes.

Prior to the induction of diabetes, the body weight of male mice was 15% greater than females (Fig. 1B). Over the 8 weeks of experimentation, nondiabetic males had a significant weight gain of 15%, whereas the body weight of nondiabetic females did not increase (Fig. 1B). After STZ treatment, male mice had an 11% loss of body weight in the first 2 weeks which did not change thereafter, suggesting that a stable diabetes had been reached. In comparison, the body weight of female mice was not significantly altered by STZ, remaining constant for the duration of the experiment. At the end of the experiment, the body weights of diabetic and nondiabetic male mice differed by 24% (*P* < 0.0001), whereas the body weights of diabetic and nondiabetic females did not differ (Fig. 1B).

Moderate hypertension was identified in nondiabetic female Nos3‐/‐ mice (SBP: 129 ± 3 mmHg) and was equivalent to nondiabetic males (SBP: 130 ± 5 mmHg). The levels of hypertension were not changed by the presence of diabetes in female mice (SBP: 131 ± 3 mmHg) or male mice (SBP: 134 ± 3 mmHg).

### Effect of sex on diabetes‐induced albuminuria and renal function impairment

**Figure 3:**
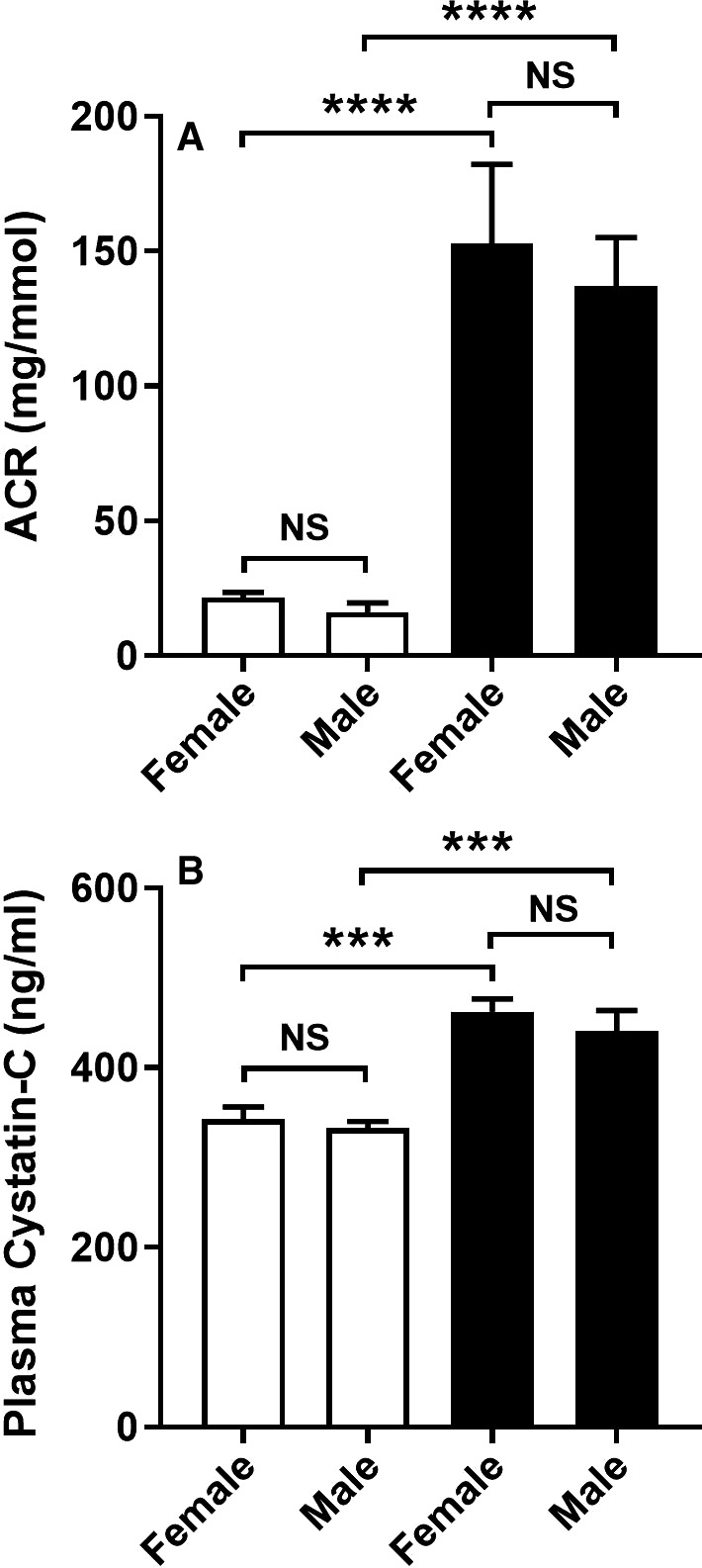
Effect of sex on albuminuria and renal function in diabetic *Nos3‐/‐* mice. At week 8 of the experiment, diabetic *Nos3‐/‐* mice (filled bars) and nondiabetic *Nos3‐/‐* mice (unfilled bars) of both sexes were assessed for their (A) urine albumin: creatinine ratio (ACR), and (B) renal function measured by plasma cystatin C levels. Data = mean ± SEM, *n* = 8. ***P < 0.001. ****P < 0.0001. NS = not significant.

**Figure 4:**
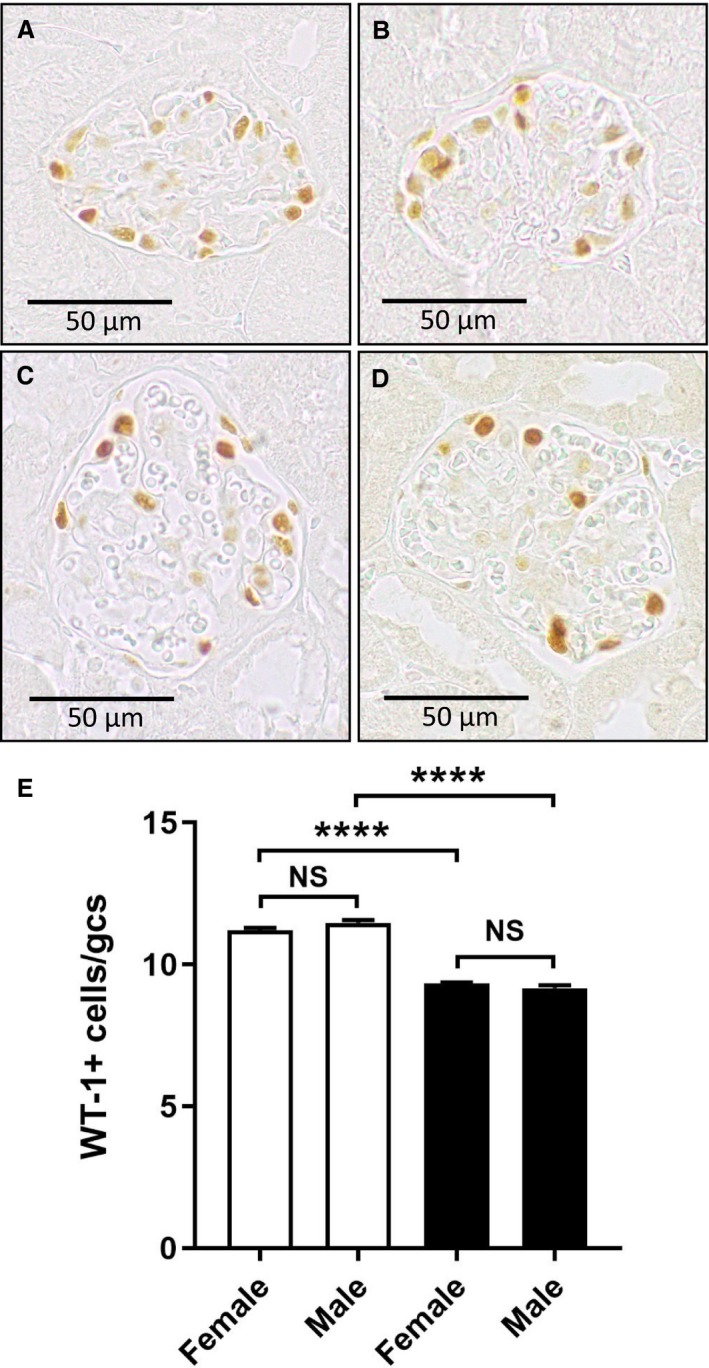
Effect of sex on podocyte loss in diabetic *Nos3‐/‐* mice. Immunostaining of WT‐1 (brown) identified podocytes in the glomeruli of (A) a nondiabetic *Nos3‐/‐* male mouse and (B) a nondiabetic *Nos3‐/‐* female mouse, which were reduced in number in (C) a diabetic *Nos3‐/‐* male mouse and (D) a diabetic female Nos3‐/‐ mouse at week 8 of diabetes. (E) A graph of WT‐1+ cells per glomerular cross‐section (gcs) demonstrates that diabetic *Nos3‐/‐* mice (filled bars) have fewer podocytes than nondiabetic *Nos3‐/‐* mice (unfilled bars), with a similar reduction seen in both sexes. Data = mean ± SEM, *n* = 8. ****P < 0.0001. NS = not significant.

Compared to nondiabetic mice, levels of urine albumin were increased eightfold in both male and female diabetic mice at week 8 after STZ and were not different between sexes (Fig. 3A). Plasma levels of cystatin C were increased by 33% in males and females at week 8 of diabetes compared to nondiabetic mice, demonstrating mild renal function impairment in both sexes (Fig. 3B). In concordance with the development of diabetic albuminuria, immunostaining of WT‐1 identified a 17–20% reduction in the number of glomerular podocytes at week 8 of diabetes which was similar for males and females (Fig. 4A–E).

### Development of diabetic kidney damage in male and female mice

Nondiabetic male and female mice had a similar kidney weight/body weight ratio (KW/BW: males 0.98 ± 0.10%, females 1.00 ± 0.06%). At 8 weeks after STZ, significant renal hypertrophy was detected by an increase in the kidney weight/body weight ratio (KW/BW: males 1.48 ± 0.08%, females 1.54 ± 0.11%; both *P* < 0.0001 vs. nondiabetic controls) which was comparable in both sexes.

**Figure 5:**
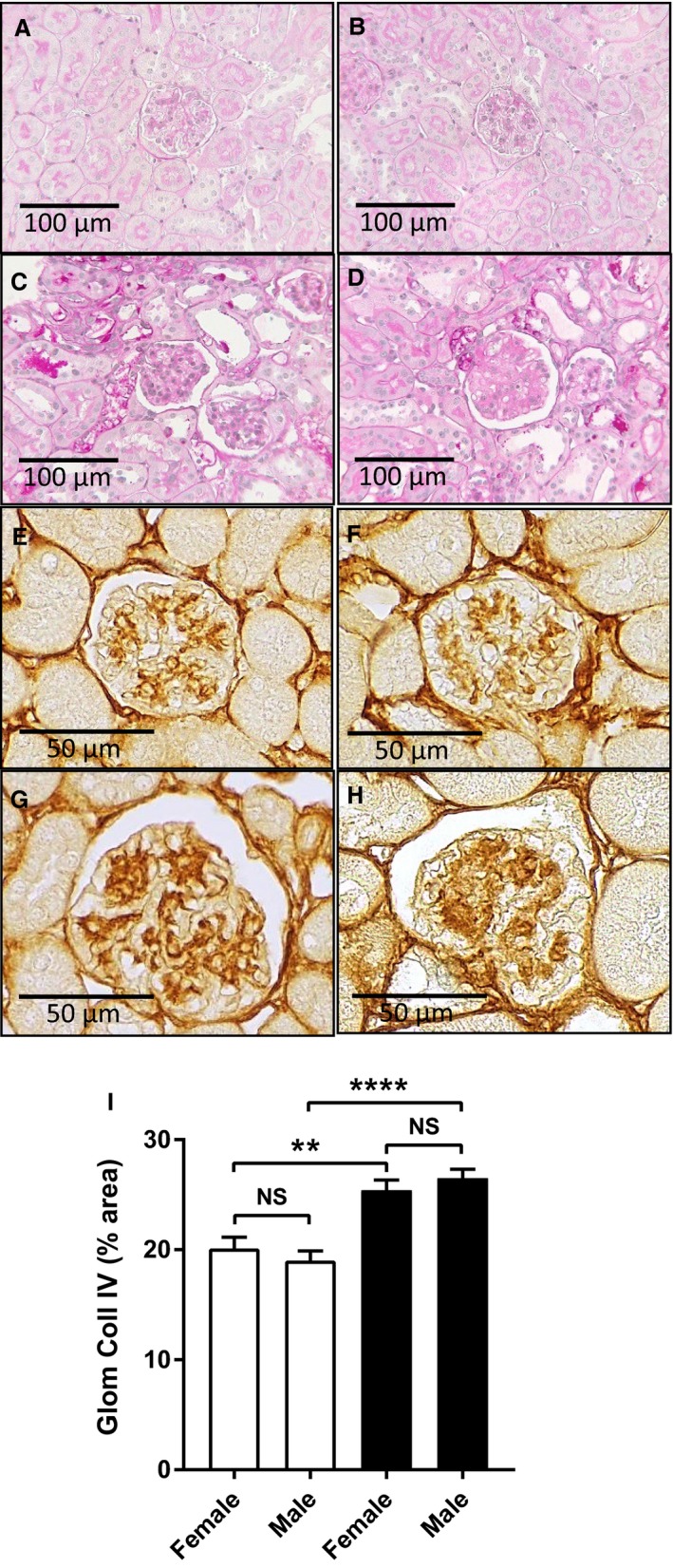
Effect of sex on diabetic kidney damage in *Nos3‐/‐* mice. (A–D) PAS‐stained kidney sections. Nondiabetic kidneys from (A) a female and (B) a male *Nos3‐/‐* mouse displayed typical normal structure at 16 weeks of age. After 8 weeks of diabetes, similar histological damage was seen in (C) a female and (D) a male *Nos3‐/‐* mouse, which included significant PAS‐stained deposits in glomeruli and dilation or atrophy in some tubules. (E–H) Immunostaining for collagen IV. Nondiabetic kidneys from (E) a female and (F) a male *Nos3‐/‐* mouse showed similar staining of collagen IV in the glomerular basement membrane. After 8 weeks of diabetes, significant mesangial deposition of collagen IV was present at similar levels in (G) a female and (H) a male *Nos3‐/‐* mouse. (I) Image analysis of the area of glomerular collagen IV staining in nondiabetic *Nos3‐/‐* mice (unfilled bars) and diabetic *Nos3‐/‐* mice (filled bars). Original magnification: (A–D) ×250, (E–H) ×400. Data = mean ± SEM, *n* = 8. ***P < 0.001. NS = nonsignificant.

Histological staining of kidneys with PAS reagent (Fig. 5A–D) showed that increased PAS deposits were present in most glomeruli of diabetic mice and that some tubules in diabetic mice were dilated or atrophic. This histological damage was similar in diabetic male and female mouse kidneys. Tubular injury was assessed by real‐time PCR of kidney injury molecule‐1 (KIM‐1), which identified a 25‐fold increase in KIM‐1 mRNA levels in both male and female diabetic mice (Table 1).

**Table 1:** Kidney expression of genes associated with injury

Gene expression	Nondiabetic		Diabetic	
	Female	Male	Female	Male
CD68 mRNA/18s	1.0 ± 0.2	1.2 ± 0.1	4.0 ± 1.0*	4.0 ± 0.8*
TNF‐*α* mRNA/18s	1.0 ± 0.3	1.7 ± 0.2	5.6 ± 1.1*	5.5 ± 1.1*
CCL2 mRNA/18s	1.0 ± 0.2	1.1 ± 0.2	8.2 ± 1.6*	8.5 ± 1.7*
KIM‐1 mRNA/18s	1.0 ± 0.4	1.0 ± 0.4	26 ± 7*	25 ± 7*
TGF‐*β*1 mRNA/18s	1.0 ± 0.2	1.1 ± 0.2	2.7 ± 0.5*	2.8 ± 0.7*
Fibronectin mRNA/18s	1.0 ± 0.2	0.4 ± 0.1	3.3 ± 0.7*	3.4 ± 1.2*
Collagen I mRNA/18s	1.0 ± 0.2	0.6 ± 0.2	5.0 ± 1.2*	4.0 ± 0.9*
Collagen IV mRNA/18s	1.0 ± 0.2	0.8 ± 0.1	2.1 ± 0.3*	2.2 ± 0.5*

Data = mean ± SD. *n* = 8 for all groups.

* *P* < 0.0001 versus nondiabetic control by ANOVA.

Immunostaining showed increased deposition of collagen IV in diabetic glomeruli compared to nondiabetic glomeruli, indicating mild to moderate glomerulosclerosis (Fig. 5E–H). Quantitative image analysis found that the glomerular area with collagen IV deposition increased by approximately 30% in diabetic compared to nondiabetic mice (Fig. 5I). This increase in glomerular deposition of collagen IV was comparable in both male and female diabetic mice (Fig. 5I).

Real‐time PCR analysis found elevated levels of mRNA for markers of renal inflammation (CD68, TNF‐*α*, CCL2) and renal fibrosis (TGF‐*β*1, fibronectin, collagens I and IV) in diabetic compared to nondiabetic kidneys, which were not different in male and female mice (Table 1).

## Discussion/Conclusion

Given the growing prevalence of DKD and the urgency for developing effective treatments, there is considerable importance in recognizing potential sex differences which could influence disease progression and therapeutic strategies. Our study has shown that female and male *Nos3‐/‐* mice of equal age and with equivalent diabetes and hypertension have a similar onset of diabetic renal injury in terms of albuminuria, renal function impairment, inflammation, podocyte loss, glomerulosclerosis, tubular damage, and kidney fibrosis. This finding has important implications for the future use of this model in studies of DKD.

Our study has identified a strategy for achieving similar levels of diabetes in male and female *Nos3‐/‐* mice. As previously shown (Tesch et al. 2015), male mice develop significant diabetes at week 1 after the fifth injection of STZ (55 mg/kg/day), which subsequently reaches a stable plateau at week 3. In comparison, female mice are more resistant to developing diabetes, but will achieve a similar level of diabetes as males from week 3 if they are given an extra (sixth) injection of STZ at 1 week after the fifth injection. Indeed, our ability to achieve equal diabetes in male and female mice is supported by the measurement of HbA1c levels at week 8, which show no difference between sexes. This finding opens up the opportunity for future studies to compare the development of diabetic complications in male and female *Nos3‐/‐* mice.

Diabetic *Nos3‐/‐* mice are widely used for studies of DKD (Takahashi and Harris, 2014). We have previously shown that week 8 of diabetes in male Nos3‐/‐ mice is an excellent time for beginning intervention studies with a novel therapy, given that many of the elements of DKD, including albuminuria, renal function impairment, and glomerulosclerosis, are already established (Tesch et al. 2015). Furthermore, we have shown that specific targeting of disease mechanisms from week 8 can inhibit or halt the subsequent progression of DKD in this model (Tesch et al. 2015). Our current finding, showing equal diabetic renal injury in males and females at week 8 after STZ‐induced type 1 diabetes, suggests that future intervention studies can be expanded to include both sexes. This finding is also supported by a recent report showing that male and female *Nos3‐/‐ db/db* mice with equivalent type 2 diabetes have similar renal injury (Ma et al. 2019).

There are multiple advantages of using both sexes in studies of DKD. Firstly, it allows for direct sex comparisons in the development of specific disease mechanisms over time. Secondly, it facilitates the determination of sex‐specific responses to treatments. Thirdly, it improves the logistics of DKD studies by reducing animal breeding and experimental time. This is particularly important in colonies of *Nos3‐/‐* mice which have reduced litter sizes (Drazen et al. 1999).

There are some limitations in interpreting the results of this study. Our study compared the development of DKD at a single time‐point and it is possible that the progression of DKD may vary over time in males and females, particularly as hormone levels change with age. However, our previous research has shown that intervention studies can be performed over a relatively short period (weeks 8–15 after STZ) in this model with measurable outcomes (Tesch et al. 2015). Based on our current findings, we expect that if male and female *Nos3‐/‐* mice are matched for age, hypertension, and diabetes, the development of DKD is likely to progress similarly in this time period and perhaps longer. Another limitation of our study is that we assessed DKD before the development of substantial tubulointerstitial fibrosis, which is seen after 24 weeks of diabetes in *Nos3‐/‐* mice (Stangenberg et al. 2018), and so we cannot ascertain whether tubulointerstitial fibrosis develops similarly in male and female *Nos3‐/‐* mice. Our real‐time PCR results show a comparable increase in mRNA levels of KIM‐1 and profibrotic molecules in the whole kidneys of male and female *Nos3‐/‐* mice at week 8, indicating that tubular injury and kidney matrix production are similar in both sexes at this time, and may progress similarly with longer diabetes.

In conclusion, we have demonstrated procedures to obtain equal levels of type 1 diabetes in male and female *Nos3‐/‐* mice which result in the equivalent onset of DKD in these mice. This finding will enable future studies in this model to incorporate both sexes and thereby provide a more comprehensive outcome when examining the impact of novel intervention therapies in DKD.

## Conflict of Interest

The authors have no conflict of interest to declare.

## Statement on Ethics

All animal experiments conform to internationally accepted standards. These experiments were approved by the Monash Medical Centre Animal Ethics Committee and were conducted in accordance with the Australian Code of Practice for the Care and Use of Animals for Scientific Purposes, 8th edition (2013).
